# A Survey and Ontology of Blockchain Consensus Algorithms for Resource-Constrained IoT Systems

**DOI:** 10.3390/s22218188

**Published:** 2022-10-26

**Authors:** Misbah Khan, Frank den Hartog, Jiankun Hu

**Affiliations:** School of Engineering and Information Technology, University of New South Wales, Canberra, ACT 2600, Australia

**Keywords:** blockchain, internet of things, consensus algorithms, consensus ontology

## Abstract

The basic properties of blockchain, such as decentralization, security, and immutability, show promising potential for IoT applications. The main feature—decentralization of blockchain technology—depends on the consensus. However, consensus algorithms are mostly designed to work in extensive computational and communication environments for network security and immutability, which is not desirable for resource-restricted IoT applications. Many solutions are proposed to address this issue with modified consensus algorithms based on the legacy consensus, such as the PoW, PoS, and BFT, and new non-linear data structures, such as DAG. A systematic classification and analysis of various techniques in the field will be beneficial for both researchers and industrial practitioners. Most existing relevant surveys provide classifications intuitively based on the domain knowledge, which are infeasible to reveal the intrinsic and complicated relationships among the relevant basic concepts and techniques. In this paper, a powerful tool of systematic knowledge classification and explanation is introduced to structure the survey on blockchain consensus algorithms for resource-constrained IoT systems. More specifically, an ontology was developed for a consensus algorithm apropos of IoT adaptability. The developed ontology is subdivided into two parts—CONB and CONIoT—representing the classification of generic consensus algorithms and the ones that are particularly proposed for IoT, respectively. Guided by this ontology, an in depth discussion and analysis are provided on the major consensus algorithms and their IoT compliance based on design and implementation targets. Open research challenges and future research directions are provided.

## 1. Introduction

Blockchain and IoT are considered digital transformation technologies. The blockchain is a secure and decentralized ledger. The most important characteristic of blockchain is decentralization. Decentralization on the blockchain is achieved using consensus algorithms, implying that no single authority can decide on the network, but everyone has consent and the final decision cannot be made without their votes [1]. This sense of authority cannot only increase trust in the system but also ensure widespread adoption of the technology. On the other hand, the IoT is also a very competitive technology and is part of (or going to be part of) almost every big industry, such as smart homes [2,3,4,5,6,7,8], construction [9,10,11], smart grids [12,13,14,15,16,17,18,19,20,21,22,23], automated car manufacturing industries [24,25], supply chains [26,27,28,29,30,31,32], healthcare [33,34,35,36,37,38], the space industry [39,40], etc. If every industry is involved in IoT, then you may end up giving control of your home or health records (or any other domain) to a centralized authority, this will raise the issue of the central point of failure and place your total trust in this authority [41]. On the other hand, if the authorities are public, then there are some issues, such as privacy and the leakage of personal data [42,43]. To overcome these issues, the blockchain concept has been proposed as a solution [41] due to its decentralized but private nature. The main features of the blockchain are briefly explained below. (1) Decentralization: Various consensus algorithms, such as the PoW (Bitcoin, Ethereum 1), PoS (Ethereum 2), BFT (Hyperledger), directed acyclic graph(s) (DAG) (IOTA, Hashgraph, Byteball, Nano), etc., are proposed to ensure the decentralization feature. They are designed so that everyone on the network could add some input to the network in terms of energy, stake, or reputation, together with some fair-play control mechanisms. (2) Immutability: This property is insured by replicating and distributing cryptographically linked data ledgers across a peer-to-peer network using hashing techniques, e.g., SHA256. By design, immutability requires a lot of data storage, since every peer on the network is storing a complete copy of the data or at least a fair amount of data required to verify the integrity of the ledger. (3) Privacy: Privacy is ensured by using encryption/decryption based on asymmetrical key pair values, i.e., public and private keys for every user. The privacy preserving techniques, such as Rivest–Shamir–Adleman (RSA), elliptic curve Diffie–Hellman exchange (ECDHE) and elliptic curve cryptography (ECC) ensure that only an authorized user can access the actual state of the data, and the remaining network will only store encrypted data strings. (4) Autonomy: Using distribution and decentralization techniques, the devices can communicate with each other without the convenience of servers being present. In particular, IoT systems can benefit from this characteristic to decouple applications and make them device-agnostic. Furthermore, blockchain provides distributed open ledgers where devices can query the trusted information using self-executable programs (smart contracts) [44].

Other features are by-products of blockchain, such as traceability, autonomy, integrity, anonymity, smart contracts, etc. By closely analyzing, these techniques work perfectly for applications, such as finance or other applications that have (or are willing to provide) the resources for the required extensive computing power, storage, and a stable network. However, the IoT systems are limited on resources, such as storage, computational power, and energy; thus, blockchain cannot be implemented as it is [45]. Therefore, the variation and optimization of blockchain technology are required to make it work with resource-constrained devices. Some achievements have been made in the field, focusing on decentralization (consensus) by optimizing legacy consensus algorithms [46,47,48,49,50], together with other solutions, such as optimal storage management [51] and edge computing [52,53].

### 1.1. Motivation and Related Work

To demonstrate the motivation of our work, we provide a summary of related surveys on consensus algorithms in Table 1. The article [54] presented a taxonomy of the evolution of the consensus algorithm from PoW to PoS. Furthermore, it classified the field knowledge with respect to the aspects of origin, design, performance, and security. However, this work missed most of the third-generation consensus algorithms and focused on generic blockchain applications rather than IoT. Another study [55] provided a comprehensive analysis of blockchains in cyber-physical systems and discussed the consensus mechanisms used for Industry 4.0. Moreover, it also discussed third-generation consensus algorithms, such as Raft, Paxos, etc. It highlighted future directions and discussed the role of blockchain and consensus in IoT. Another work [56] presented a survey on consensus algorithms and discussed the requirements of the IoT resource environment for implementation. Furthermore, it presented a thorough analysis of legacy and third-generation methods with respect to latency throughput and mentioned directed acyclic graph (DAG) consensus for IoT as well. However, this work did not provide a comparison of these consensus methods and did not include adaptability or IoT applications. Another survey [57] analyzed consensus algorithms and presented a framework to evaluate consensus mechanisms applicable to legacy and nonlinear consensus algorithms. However, the focus of this study is not on resource-constrained IoT systems. Ref. [58], discussed IoT and blockchain in terms of IoT architectures and applications. They also discussed blockchain consensus methods with examples of IoT applications, including the DAG protocol. Ref. [59], presented a review of blockchain-based IoT in terms of security, robustness, and self-maintenance. They discussed how a blockchain can transform the cloud-centered IoT environment. Furthermore, they discussed general consensus algorithms, which did not include any third-generation consensus method or DAG. In general, these existing surveys are based on an intuitive classification of domain knowledge, making it difficult to reveal the intrinsic logical connections between knowledge concepts in the field. This paper aims to overcome this limitation by providing an ontology-guided survey on blockchain consensus algorithms for resource-constrained IoT systems. Ontology is a powerful tool that could automatically classify a set of concepts in a subject area and display their properties and the relations between them. The ontology can also help computing machines better understand the relevant concepts and process relevant information, which is very useful for practical industry applications.

### 1.2. Main Contribution

As stated in Table 1, this paper presents an ontology-guided comprehensive survey of blockchain consensus algorithms with respect to an IoT resource-constrained environment. Specifically, we developed an ontology and classified different consensus mechanisms based on their logical implementation details. The main contributions of this paper are as follows.

- A novel consensus ontology was developed with the help of the open-source tool Protégé. This ontology consists of two major parts: CONB.owl and CONIoT.owl. CONB is the main ontology, which is used to map five categories of consensus—competitive, comparative, vote-based, non-linear, and collaborative.
- A subclassification of the CONB.owl Ontology is provided. Subclasses consist of consensus algorithms based on their implementations and the global state finality decision process, i.e., computational power, stake, Byzantine agreement, and collaboration of more than one consensus for the finality decision process or non-block structure. In addition, the IoT_Adaptability subclass was created, which is featured with properties of IoT_Friendly, Not_IoT_friendly, and partially_IoT_Friendly in the main CONB.owl hierarchy, which is used to pair every consensus algorithm with an associated subclass.
- We advanced the CONIoT.owl ontology as a partial extension of comparative and non-linear classes of CONB.owl ontology. CONIoT.owl consists of Po* and DAG consensus methods.
- An ontology-guided comprehensive survey is provided on blockchain consensus algorithms for resource-constrained IoT Systems. Discussions are provided on the limitations of existing consensus mechanisms for IoT environments and their adaptability to IoT. Future research directions are provided. To our knowledge, this is the first ontology-guided survey in the field.

The rest of the paper is organized as follows. In Section 2, an overview of blockchain and consensus for IoT is presented. In Section 3, a consensus ontology is introduced, explaining the class hierarchy of CONB and CONIoT. In Section 4, the main challenges of blockchain and IoT adaptability and future research directions are discussed. Lastly, Section 5 presents the concluding remarks of this paper.

## 2. An Overview of Blockchain and Consensus for IoT

### 2.1. Blockchain Evolution

**Figure 1:**
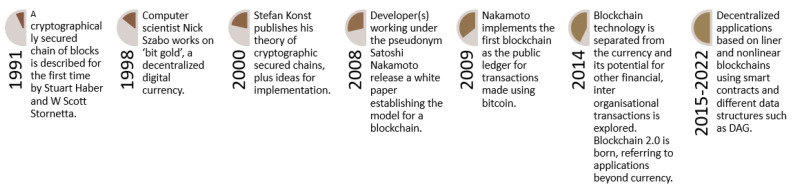
Evolution of blockchain technology.

Bitcoin is incorrectly perceived as having coined the blockchain concept. However, the idea was discussed years prior. In 1991, two mathematicians proposed a similar solution to implement a system where documented time stamps could not be tampered with [60]. In 1998, cryptographers and computer scientists proposed the idea of solving a puzzle tailored to computing power, and introduced the idea of smart contracts [61], which are similar to the current concepts of power of work (PoW) and smart contracts adopted in the blockchain. Later, in 2000, a German computer scientist proposed the idea of cryptographically linked blocks that can be traced back to the genesis block. In 2008, a developer or organization operating under the pseudonym Satoshi Nakamoto published a white paper instituting the model for a blockchain and the same organization implemented the first decentralized financial system as Bitcoin [62] using the PoW as a consensus mechanism. The PoW by design requires high computational power and transactional delay (10 min and difficulty level changes after 2016 blocks) for security (chain split) and optimal data storage (Moore’s law). In the initial years, bitcoin was the only cryptocurrency available, but later, in 2014, Ethereum separated the blockchain from the sole idea of cryptocurrency and introduced smart contracts as a programmable logical layer to implement decentralization to other applications as well [63]. Ethereum (at the start) was also based on the PoW consensus mechanism, although it modified the mining process by altering the difficulty level after each block (rather than 2016), and added an intended delay between 10 and 19 s. If a new block falls under this threshold, then the difficulty level will remain the same; otherwise, it will increase or decrease the difficulty level accordingly. Furthermore, with the reduced difficulty level (and to ensure security), Ethereum used the GHOST protocol to deal with split chains (Forks) by rewarding UNCLE blocks as well. Later, Ethereum was partially shifted to proof-of-stake (PoS) consensus with multiple techniques, such as sharding, for optimal data traceability and storage. The evolution of blockchain is demonstrated in Figure 1.

### 2.2. Blockchain Generations and Consensus Evolution for IoT

**Figure 2:**
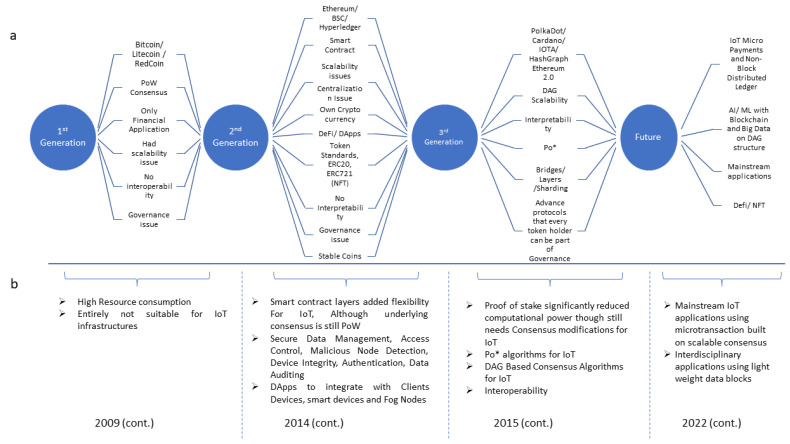
(**a**) Blockchain generations; (**b**) consensus evolution along generations.

Although the idea of blockchain has been discussed before, Bitcoin was the first successful application accepted by the public. Therefore, Bitcoin (using the traditional PoW consensus algorithm) is classified under the first-generation blockchain. Besides Bitcoin, other blockchains exist, such as Litecoin and Redcoin, which have been forked on the Bitcoin open-source code. These first-generation blockchains were solely built for financial applications; they work on the PoW consensus, which requires high computational power, specialized mining hardware (rigs), and high data storage for data replication on every node. Furthermore, these blockchains do not have interoperability and governance, resulting in a separate mining network from the actual users of the applications. These characteristics of the first-generation blockchains make them highly inappropriate for IoT environments; thus, they cannot be used in mainstream IoT applications. Moreover, second-generation blockchains started with Ethereum; other blockchains (such as Hyperledger or BSC) have the same idea. These blockchains have inherited the idea of decentralization but introduced the concept of logical/conditional programmable contracts (known as smart contracts, chaincode, etc.). The second-generation blockchain was built on the updated PoW or BFT, which is a vote-based consensus mechanism, and has a centralization issue. BFT only works well with small or closed networks, while in large networks, the BFT performance decreases and the scalability issue looms due to the voting and communications overhead. Consequently, these blockchains are not well-suited for IoT applications. Nevertheless, the second-generation blockchain has found that some IoT applications, such as secure data management [64], access control [65], malicious node detection [66], device integrity [67], authentication [68], data auditing [62], etc., based on smart contracts; however, underlying consensus algorithms are inefficient at being implemented in a resource-constrained environment. Additionally, the second-generation blockchain is mostly implemented as DAPPs, using fog nodes or edge computers as intelligent devices to work with IoT systems. The third-generation blockchain is designed to overcome the consensus-based issue of previous blockchains. It introduced various consensus protocols, including PoS (to be discussed in detail in our proposed ontology later), which addresses the energy consumption issue suffered in the first- and second-generation blockchains. Moreover, these algorithms were built on different parameters that apply to different applications. Although not all these consensus algorithms can be implemented in IoT; there exist a few algorithms that are specifically designed for IoT environments, such as Po* and DAG, which can drastically improve the scalability. Additionally, the third-generation blockchain is built on performance optimization techniques, such as sharding, layers, and bridges to improve interoperability. The evolution of consensus algorithms has radically improved the practicality of blockchains for IoT applications. The blockchain generation and evolution of consensus for IoT are presented in Figure 2. Consensus algorithms of these blockchain generations will be discussed and analyzed in detail in accordance with the consensus ontology proposed in the next section.

## 3. Consensus Ontology

Consensus is an integral part of blockchain-based systems. IoT networks consist of resource-constrained devices, while many consensus algorithms have a high resource demand in terms of computation power, storage capacity, and communication overhead. Hence, many efforts have been made to adapt the legacy consensus methods to the IoT setting. This phenomenon has significantly increased the challenge of developing a suitable ontology, as it has to cover the legacy algorithms and it also needs to introduce new data structures and a finality process (consensus for the global state) in accommodating the adaptability towards the IoT applications. This section covers the consensus ontology for IoT systems in two perspectives; two ontologies were created with a formal representation in RDF/OWL format, which can be downloaded here (CONB, CONIoT). First, a consensus ontology for blockchain (CONB) was developed to cover the consensus classification in general blockchain systems. Second, the consensus ontology for IoT (CONIoT) was developed to represent the consensus algorithms proposed for specialized IoT networks considering the resource limitations. CONIoT is an extension of the basic consensus classification presented in CONB (Proof of * and DAG).(Source). Both ontologies were built using a top-down approach, starting from original consensus algorithms to the variations or optimized versions of those algorithms for IoT Networks. This paper provides the mapping of existing consensus algorithms in the literature, as well as a comparison methodology for subclasses of consensus methods and ObjectProperties linked with the applications. This mapping provides a structured guide to analyze the targeted intention for a particular use case. We propose creating ontologies instead of simple taxonomies for various reasons. First, a taxonomy would be very complex as we have defined many different entities and the relationships between the entities (the “properties”) are diverse and, thus, difficult to capture in a single flat taxonomy. Representing consensus algorithms as a coded ontology allows the reader to view various aspects of the ontology in isolation, depending on which aspect is of interest at a given time, using specialized software tools, without losing the overall consistency. Second, a coded ontology makes it easier for future researchers and collaborators to use it for their own work, to expand or change the ontology as required, and to link it to other related ontologies of interest. Therefore, ontology engineers have developed various best practices and standards that simplify reuse, expansion, and linking [69]. We acknowledge that our ontologies do not follow all of these practices. A concrete example concerns the use of upper ontologies. We do not provide explicit references to foundational upper ontologies as, in our opinion, introducing these ontologies at the first release of CONB would have complicated its understanding and, consequently, its adoption by the IoT and blockchain industry.

### 3.1. CONB

**Figure 3:**
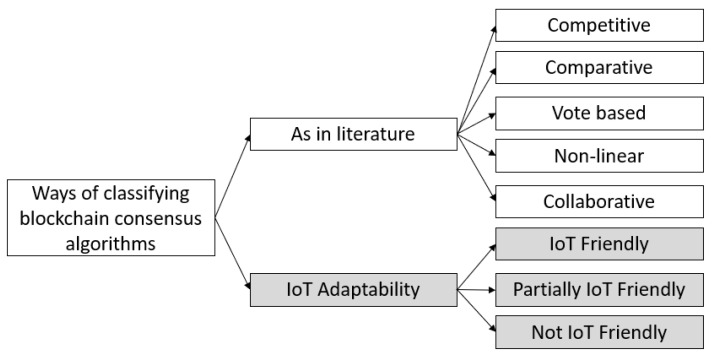
Proposed consensus classification.

Blockchain is built on the concept of decentralization, a trustless network where anyone can participate; it can be either an honest or malicious node. The security and robustness of a blockchain network depends on its underlying consensus algorithm. The PoW is considered the most well-known and widely adopted consensus method for blockchain. Due to the intended high computations to ensure the security and storage requirement, it is mostly used in financial applications, i.e., Bitcoin or ETH. Gradually, as the blockchain starts becoming as attractive for mainstream applications (such as IoT, which is the focus of this study), many consensus algorithms have been introduced. The CONB presents those algorithms and uses ObjectProperties as reasons for why they are suitable (or not) for IoT networks. We classified existing algorithms reported in the literature into five classes, *Consensus_Algorithms* (1) competitive, (2) comparative, (3) vote-based (4) non-linear, and (5) collaborative. CONB represents the other subclasses and uses its ObjectProperties to determine the suitability of these algorithms in IoT systems. Furthermore, in Consensus_Algorithms, we listed IoT_Adaptability as three subclasses IoT_Friendly, Partially_IoT_Friendly, and Not_IoT_friendly, representing the degree of each consensus algorithm for general IoT systems, and linked the ObjectProperties of subclasses using domain and range descriptions. Moreover, the additional explanation of concepts is added as rds:comments under annotations. The proposed classification and the degree of adaptability to IoT is illustrated in Figure 3, and discussed in further detail below.

#### 3.1.1. Competitive

Competitive is the subclass of *Consensus_Algorithms* under the blockchain class of the main *Owl:Thing* hierarchy in CONB. Competitive consensus can be explained as a method where multiple network participants start solving the same problem at the same time; after a particular interval, only a single decision is accepted/rewarded while other solutions are deemed invalid even if they meet the criteria.

##### Proof of Work [63]

PoW is a computationally expensive algorithm due to its underlying puzzle-solving technique based on the SHA-256 hashing, which is a fixed-256Bit string. This technique is used to append a new block to the blockchain ledger; this process is called mining, and the participants of this method are known as miners. The block consists of a nonce, transactions, block ID, previous block hash, and timestamp. Miners try different nonce values to find the 256-bit hash that must be lower than (leading zeros) the difficulty set by the network, which is set after every 2016 blocks. As soon as a miner calculates the hash, he forwards it to the network. When the network receives a new block, it verifies it, stops solving the same block, and then moves to the next round. It is crucial that all miners verify the new block before moving to the next round. Otherwise, they will build up new blocks on top of an unverified bock (fork), which will certainly be rejected by other miners (if not valid). As a result, miners will waste resources (electricity and time) while the longest chain (Valid blocks) become a part of the main ledger. The practice of finding the exact nonce to meet the target difficulty is cumbersome, mathematically hard, and requires a brute-force search using specialized hardware. On the other hand, the immutability of data in the PoW is achieved by replicating the ledger over every node in the network with continuous consistency checks.

##### $IoT\_Adaptability\overset{}{\to }Not\_IoT\_friendly$

The PoW by design consists of intended delays (transaction latency), making it highly incompatible with IoT networks. Second, the storage requirement for ledger replication is another significant hindrance in IoT_Adaptability. Finally, the special hardware required for solving the puzzle makes it decidedly unsuitable for IoT networks.

##### Proof of Capacity (PoC)

PoC is built on the lines of PoW. Instead of using large computational power, this algorithm depends on the storage capacity provided by the miner. A miner stores huge chunks of data known as PLOTS. As large data PLOTS are stored by the miner, its chance of mining the new block and winning the reward increases. Since PoC eliminates the need for ASICs, this makes it more energy efficient, and the average time to mine a new block is reduced from 10 min (PoW) to 4 min.

##### $IoT\_Adaptability\overset{}{\to }Not\_IoT\_friendly$

PoC reduces the transaction delay by 60%. However, it is still not scalable enough for IoT applications. Furthermore, storing PLOTS on IoT devices is impossible considering the limited (or almost no) storage capacity of IoT devices.

##### Proof of Elapsed Time (PoET) [70]

PoET was designed with the intention to ease computational requirements and improve scalability (high throughput, low Latency). It works the same as the PoW in the sense that the miners must solve a mathematical puzzle. The selection of the next block does not depend on the competition between the miners. Instead, the miners are randomly selected based on their waiting time. The fairness of randomness is ensured by trusted execution environment(s) (TEE), where miners set their times randomly; the miner whose time expires first mines the next block and wins the block reward.

##### $IoT\_Adaptability\overset{}{\to }Not\_IoT\_friendly$

The low latency and high throughput of PoET make it suitable for IoT systems, although TEE, such as Intel Software Guard Extension(s) (SGX), make it centralized, depending on a single source of hardware provider. First, the centralized nature of the algorithm is not appropriate for IoT, as the main proposition of using blockchain is to make the network decentralized. Second, the addition of specialized hardware to the IoT network imposes system overhead. In deciding, PoET cannot be considered an IoT_Friendly consensus mechanism.

**Figure 4:**
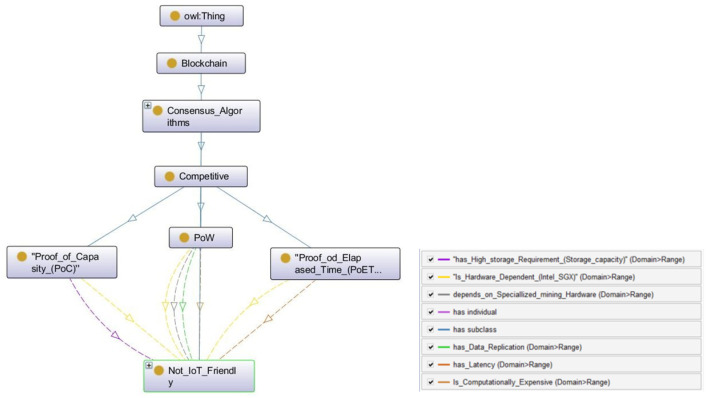
Ontology of competitive consensus class algorithms and IoT adaptability.

Based on the discussion in the competitive section, this kind of consensus algorithm is not recommended for resource-constrained IoT devices. In the competitive environment, every participant of the network tries to be as efficient as it can be in terms of computational power, storage, or time, with the aim of winning or being selected by the network to generate the new block in the ledger and winning a reward. On the contrary, an IoT system consists of many small resource-constrained devices, and every device serves the same purpose and holds the same equal local value to obtain the results. Therefore, competitive consensus belongs to Not_IoT_friendly. The ontology of competitive consensus methods is presented in Figure 4, which is a subpart of the ontology CONB.

#### 3.1.2. Comparative

Comparative consensus algorithms are presented as a subclass of *Consensus_Algorithms* in the blockchain class of the main *Owl:Thing* hierarchy. In the comparative consensus, the selection of a miner to create a new block is based on the comparison of the miner network with regard to the stake. The stake can be defined as a reprimand that a miner is willing to pay to the network in case of malicious behavior. In CONB, the comparative subclass is further divided into two subclasses—Proof_of_* (Po*) and Proof_of_Stake (PoS). Po* consists of comparative consensus algorithms, which are specialized for IoT systems, while PoS contains algorithms, which can be used in IoT networks but are not designed for it. In this section, we will only discuss the subclass PoS while Po* is discussed in detail in CONIoT.

##### Proof of Stake (PoS) [71]

PoS consensus uses validators to agree on the final state of the ledger where validators are the same as miners in the PoW. However, instead of competing, validators are selected on a lottery-based system, depending on the stake they provide in the network, which is the monetary value and signed by the digital signature of the validators. The bigger the stake, the higher the chance to create the new block. As with the PoW, every validator is bound to verify the new blocks. Otherwise, they will face a penalty (losing stake). PoS block rewards are only transaction fees associated with the transaction in each block. Nevertheless, the validator penalties can be used to incentivize good behavior. Owing to this block creation mechanism, the PoS is considered as energy “efficient-No” computational overhead, low entrance “barriers-No” special hardware requirement, comparatively “scalable-No” intended delays, and easily “extensible-part” interpretation. There should be no space on either side of a hyphen/en dash/em dash of the third-generation blockchains, and can easily be optimized with coding (smart contracts, chaincode, etc.). However, there is a flaw in the PoS design, which is known as the “Nothing-at-stake” problem. As validators are selected on a lottery basis, if the algorithm selects a validator with a low stake value, and that validator is a malicious node, then, it will append a new malicious block in the ledger and win the transaction fees (more than his stake in the network) while he has nothing or almost nothing at risk.

##### $IoT\_Adaptability\overset{}{\to }Not\_IoT\_friendly$

PoS is an improved version of the PoW consensus regarding throughput, energy efficiency, and latency, which makes it favorable for IoT networks. However, “Nothing-at-stake” is a known issue. The primary concern of the wide IoT_Adaptability is the monetary concept, and an IoT network cannot be designed on this kind of stake. Therefore, PoS is defined as a No_IoT_friendly algorithm.

##### Delegated PoS (DPoS) [72]

DPoS can be defined as a consensus of representative democracy. The ledger finality is achieved by the witness and the delegates are selected by the stakeholders of the network using Voting instead of a lottery. Witnesses are the validators who create the new blocks, and delegates can be defined as managers of the validators and block generation mechanism by defining block size, validator rewards, or network fees. The stakeholders’ voting takes place periodically (for each block, 21–100 delegates) at a rate of “one vote per share per witness”, which means that the stakeholders do not create new blocks but select witnesses and delegates to perform their work. ’Stakers’ withhold their share in the staking pool. If delegates from their staking pool create a new block, then the block reward will be shared with the ’stakers’: the bigger the stake, the bigger the reward. If selected, nodes do not participate in the network fairly, the stakeholders will replace them in the next vote.

##### $IoT\_Adaptability\overset{}{\to }Not\_IoT\_friendly$

DPoS inherits all of the positive points of PoS, and are in fact more scalable. However, the ’stakers’, witnesses, and delegates make the network more centralized. Furthermore, DPoS is also monetary value-dependent. Hence, the concept of staking pools is highly incompatible with IoT networks.

##### Leased PoS (LPoS) [73]

LPoS works on the lines of PoS, and the main purpose of the LPoS is to solve the centralization problem of the PoS and PoS variants. As in PoS, only the wealthier party (higher stake node) has more chances to create a new block, while in LPoS, the nodes with lower stakes can lease/borrow some stakes from nodes with higher stakes. The borrower does not own the stakes, which are still linked with the original wallet. However, after leasing, the borrower will have a greater chance of being selected in the block generation. Moreover, if the borrower succeeds in creating the block, then the reward is divided between both the borrower and the node with a high stake.

##### $IoT\_Adaptability\overset{}{\to }Not\_IoT\_friendly$

LPoS has solved the centralization issue, leading to a more decentralized system. However, the monetary values are still used in incentivizing the miners, which makes it unsuitable for IoT Networks.

##### Proof of Burn (PoB) [74]

Burning tokens in the blockchain affect the price of the cryptocurrency. Cryptocurrencies are built on supply and demand models. When the supply decreases, the demand to be met will increase, and vice versa. PoB is built on the same models. Unlike PoS where a user puts some value on the stake, PoB nodes burn some of their tokens (sending tokens to irreversible addresses), the more tokens a node burns, the more chances it has to create a new block and earn a reward.

##### $IoT\_Adaptability\overset{}{\to }Not\_IoT\_friendly$

This approach can be beneficial to financial applications, which run on token scarcity. However, this consensus model cannot be efficiently implemented in IoT applications because the IoT does not work with monetary values.

##### Proof of Importance (PoI)

PoI also works similarly to PoS, and its intention is the same as LPoS to make the system more centralized. Unlike favoring the nodes with high stakes, PoI also considers other parameters in selecting the validator of the next block, such as vested tokens, net transfer, and clustering. This mechanism is built on network clustering and page-ranking protocols, which favor the higher participation of the nodes in the network rather than just monetary value. Moreover, the importance score is also associated with the winning node, which discourages hoarding (ends up having a low importance score).

##### $IoT\_Adaptability\overset{}{\to }Not\_IoT\_friendly$

The goal of PoI is to provide fairness in the validator selection process. It still uses vested tokens, net transfer, and clustering parameters, which are not fitting for IoT networks or IoT applications.

##### Casper [75]

Casper is an optimized version of the PoS proposed by the Ethereum blockchain to replace its PoW-based consensus mechanism. Casper is based on the basic concept of the PoS stakes; however, the implementation details are more complex than just staking some money to create the new block. Furthermore, Casper also provides the solution to the nothing-at-stake problem, because every validator must stake (1500 ETH) to be considered a validator. Furthermore, Casper has two versions: corrected-by-construction (CBC) and friendly-finality-gadget (FFG). FFG is implemented as a first phase in the beacon chain (Phase 1) by retaining the block generated by the PoW and adding PoS mechanisms using smart contracts. Moreover, Casper also uses the very greedy GHOST protocol. Unlike the longest chain win rule (where only the longest chain is selected and other forks are discarded), the GHOST protocol also incentivizes the UNCLE (the main forking blocks) and NEPHEW blocks (the sub-blocks of the uncle block). These incentives are not treated as main chain blocks but are considered valid if they meet the parameters defined by the network (back-seven level).

##### $IoT\_Adaptability\overset{}{\to }Not\_IoT\_friendly$

Casper provides many promising benefits in terms of security and scalability, though the implementation requires much computation, making it difficult to use in IoT systems. On the other hand, Casper is also monetary value-dependent as with all other PoS-based algorithms. So, it is deemed as Not_IoT_friendly.

**Figure 5:**
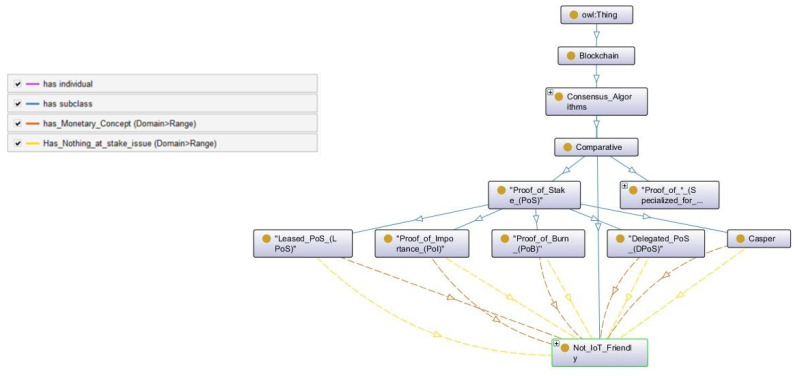
Ontology of comparative consensus class algorithms and IoT adaptability.

PoS-based algorithms have increased the scalability, system performance, security, and interoperability at significant levels. There are many other variants, such as secure proof of stake (SPoS), proof of stake boo (PoS Boo), high-interest proof of stake (HiPoS), asset PoS (APoS), tiered proof of stake (TPoS), variably delayed proof of stake (vDPoS), etc., which are generated via modifying a few parameters to satisfy particular use cases. However, none of these algorithms is intended for the IoT application. In principle, PoS can be made suitable for IoT systems since it works with smart contracts, which gives the flexibility to play around with the stake values in fitting the IoT setting. The ontology for the comparative consensus algorithms is presented in Figure 5, which only represents the sub-classes of PoS consensus, and the Po* consensus algorithms are discussed in CONIoT.

#### 3.1.3. Vote Based

Vote-based consensus involves direct voting for a new block. Unlike the PoW, where every miner competes with each other, or PoS, where every validator must ’stake’, vote-based consensus uses the Byzantine agreement to reach the consensus, which is defined as the Byzantine fault tolerance (BFT). BFT assures that the system will run smoothly even if there are a certain number of malicious nodes (fault tolerance). If the majority of nodes are malicious, then the system is susceptible to failure (51% attack). BFT-based systems can have two types of Byzantine failures. One is the fail-stop failure, which could be caused by network errors, i.e., message delivery failure or connection error, etc. The second failure is caused by the wrong behavior from an arbitrary node or a Byzantine node (malicious node), e.g., deliberately providing misleading responses. Vote-based consensus algorithms work well in small networks and, hence, are mostly used in private or consortium blockchains. These algorithms are mapped in the class *Consensus_Algorithms* in the *Owl:Thing* hierarchy of CONB. The variants of BFT are discussed in the following section.

##### Practical BFT (PBFT) [76]

PBFT forms after the BFT, and provides a replication of the Byzantine state machine. PBFT works in a sequential way, i.e., a primary node (leader) and then a secondary node (backup). The consensus starts with a client request (consensus call) to the leader node, then the leader node broadcasts it to the backup nodes. Both types of nodes process the request and send back the decision. The request is considered complete when 3f + 1 replies are received, where f is the number of faulty nodes. Leader and backup nodes can be changed after every consensus round using the view change protocol.

##### $IoT\_Adaptability\overset{}{\to }Partially\_IoT\_friendly$

Owing to the implementation details, such as not using any high computational puzzles or high stakes, PBFT could be a good fit for IoT applications. However, this algorithm works well only for small or close networks. Furthermore, there is a communication overhead (broadcasting messages) that needs to be considered when using it for IoT systems. Consequently, the PBFT is Partially_IoT_friendly.

##### Delegated BFT (dBFT) [77]

dBFT works the same as PBF. The core difference is using the delegates, which means the token holders do not take part in the decision-making but choose bookkeepers/delegates to perform the consensus. dBFT works in a hierarchical governance system.

##### $IoT\_Adaptability\overset{}{\to }Not\_IoT\_friendly$

dBFT uses delegates, which makes it less scalable than PBFT in terms of transaction throughput, and inherits the centralization issue of all BFT protocols. However, the latency is a disadvantage for IoT_Adaptability.

##### Federated BFT (FBFT) [78]

FBFT is the only Byzantine agreement that is built with the intention of decentralization. In FBFT, every Byzantine general (federates) is responsible for its own chain, and it receives the request, organizes, and establishes a truth according to its best knowledge. This version was introduced for the microfinance application in Steller and Ripple, with slightly different implementation details. Both are presented as subclasses of FBFT in CONB and discussed in the following sections.

##### Steller Consensus Protocol (SCP) [79]

SCP works with the quorum and quorum slice. A quorum involves the federates that are responsible for reaching a decision, while the quorum slice is a subset of the federate, which is responsible for a process to reach the agreement. Furthermore, the quorum slice helps in making network connections. The quorums are selected by actual token holders of the network. Consensus is achieved by nomination and balloting. Nomination starts with selecting the transactions for the quorum and asking the federates to agree on the transaction; the federates vote on the transaction, i.e., ‘I nominate Trx1’, or they can echo the votes of the neighbor federates, i.e., ‘I nominate Trx2 also’. These nomination processes are parallel, and at the end, the quorum slice is generated by taking the UNION of all nominations. Balloting is a pair (candidate, value), where the candidate is the set of transactions selected in the nomination phase, while the value is the number of votes in favor. The ballots are broadcast to the network. If a decision is made, then the consensus is complete; otherwise, it will discard the ballot and start balloting with a new pair.

##### $IoT\_Adaptability\overset{}{\to }Partially\_IoT\_friendly$

SCP is more decentralized and efficient in obtaining the final decision; therefore, this can be applied to the IoT application. However, the latency is high due to the network communication, nomination, and balloting process. Consequently, this algorithm is not perfect for IoT systems. If an IoT use case can compromise the latency, then the SCP can be partially implemented (Partially_IoT_friendly).

##### Ripple Protocol Consensus Algorithm (RPCA) [80]

RPCA is also built on FBFT, and the focus of this algorithm is to reduce transaction latency. This method works using strategist (every loyal node will agree on the same decision) and UNL (unique node list). The strategist (RPCA) runs to select the loyal nodes (not guaranteed but selected as loyal) and makes a UNL. These UNLs make the consensus and must agree on the same decision (at least 80% of all), meaning a 20% fault tolerance. If there are multiple UNLs, then at least 20% of the members should be the same, leaving 80% of nodes to accept or reject the decision.

##### $IoT\_Adaptability\overset{}{\to }Partially\_IoT\_friendly$

RPCA improves the latency enormously, which is good for IoT applications. However, the fault tolerance is only 20%. If the IoT application can ignore this fact, then this algorithm can be used in IoT systems.

**Figure 6:**
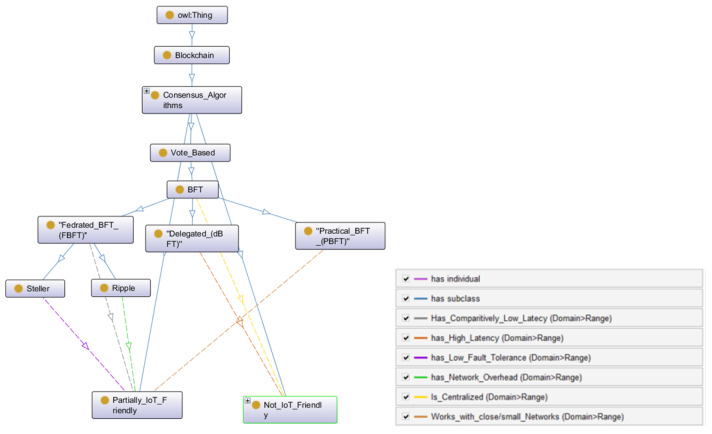
Ontology of vote-based consensus class algorithms and IoT adaptability.

Voting-based consensus algorithms are more centralized in nature, and suitable for small/close networks. The centralized nature of these algorithms makes them more efficient in terms of latency and throughput to a certain extent when compared to comparative or competitive consensus. Therefore, this can be implemented in many IoT applications, which require more closed networks and private or consortium decision-making processes, such as a smart factory, smart home, etc. The vote-based consensus ontology is presented in Figure 6, which is an instance of CONB ontology.

#### 3.1.4. Non-Linear

Non-Linear consensus is defined as an alternative to the basic blockchain structure. The blockchain is built on cryptographically connected blocks. Blocks consist of a set of transactions and other parameters, which vary from blockchain to blockchain. Then different consensus mechanisms are used to make this new block a part of the agreed blockchain state, which is propagated to the network. However, nonlinear blockchains do not follow this rule. Non-linear is a subclass of the *Consensus_Algorithms* class in CONB ontology, which is further divided into DAG and side_chains. The DAG data structure is entirely different from the conventional blockchain and highly suitable for IoT applications, as discussed in the CONIoT ontology. This section focuses on side_chains. Side chains are built on a basic blockchain structure. However, they do not work linearly as normal blockchains. Side_chains can have their own consensus protocols and have the ability to transfer assets smoothly between the chains. Furthermore, Side_chains are built on top of an underlying blockchain as a level 2 (secondary chain). Both chains work in parallel to finalize the decision on the main chain using a two-way peg and smart contracts. The two-way peg means that the asset can be transferred between both chains. However, it does not mean that the actual tokens are transferred, which will add an extra consensus and system overhead to the application. To avoid this problem, the tokens are pegged with the primary chain currency. When users make a transaction via the secondary chain, the same number of tokens are locked in the main chain. On the other hand, smart contracts are used to confirm transactions rather than going through the primary chain consensus (avoiding transaction latency). Once a new TRX is made, the smart contract will notify Mainnet about the event. As soon as TRX is verified by the secondary and Mainnet smart contracts, users can transfer assets or funds between two chains.

##### DFINITY [81]

DFINITY is a bridge that works on top of the Ethereum blockchain. DFINITY can work with any underlying consensus algorithm, such as a PoW, PoS, or even BFT. The main concept of the second-generation blockchain is “Code is the Law”; DFINITY rewrites that as “AI is the Law”, suggesting that the second layer on the existing chains can revolutionize the utility. Moreover, DFINITY works on smart contracts as the basic concept of the Side_chains, and uses the two-way peg mechanism for asset transfer.

##### $IoT\_Adaptability\overset{}{\to }Not\_IoT\_friendly$

Although DFINITY provides enormous scalability as compared to the legacy consensus methods, the transaction latency is not suitable for IoT applications. Resource-restricted applications cannot install both ledgers (primary and secondary). Moreover, running smart contract specialized nodes is required, such as the EVM.

##### Proof of Activity (PoA) [82]

PoA was proposed to extend the PoW consensus of Bitcoin using PoS consensus. PoA aims to make Bitcoin more secure by adding a PoS layer, making attacks more expensive than attacking just the PoW. Additionally, other properties are inherited with the overlay consensus mechanism, such as low latency, improved network topology, low energy consumption, and fewer transaction fees. PoA can also be considered a collaborative consensus method. Therefore, it is also listed under the collaboration class in the hierarchy *Owl:Thing*.

##### $IoT\_Adaptability\overset{}{\to }Not\_IoT\_friendly$

**Figure 7:**
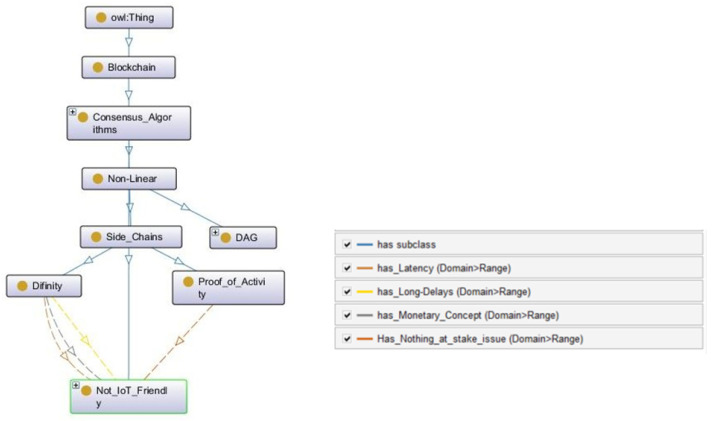
Ontology of non-linear consensus class algorithms and IoT adaptability.

PoA has improved the scalability and energy consumption issues of legacy blockchains. However, the transaction confirmation time and PoS-based staking and penalties are still not optimized enough to be used in IoT-based applications. Side_chains play an important role in improving the overall finality of the blockchain ledger. Moreover, these solutions are only implemented for existing blockchains, such as Bitcoin (PoA) or Ethereum (Polygon/Matic). Therefore, IoT adaptability is not possible, at least with the current version of side_chains. However, the use of smart contracts gives freedom to implement main-chain currencies in IoT management apps, such as micropayments on the IoT app level (where users do not need to complete the transactions on the runtime with the side_chains confirmation). There can be many other use cases and open-end future directions. Side_chains ontology is presented in Figure 7.

#### 3.1.5. Collaborative

Collaborative consensus works with the collaboration of two or more consensus algorithms. Unlike side_chains, collaborative consensus does not necessarily create an overlay on an existing chain. However, they consist of more than one consensus method on a single chain to reach the final decision. For example, they use the PoW puzzle with relaxed energy consumption for security and PoS variants to achieve consensus in a single application. Collaborative consensus is the last in the *Consensus_Algorithms* and *Owl:Thing* hierarchy of the CONB ontology.

##### Tendermint [83]

Tendermint is a combination of PBFT and PoS. Unlike PBFT, where every node must participate in the consensus, Tendermint is a permissioned method. The decision of Tendermint is finalized in two steps: pre-vote and pre-commit. Validators, similar to PoS, propose block and BFT voting be used to accept or reject the new block. The final decision is made after 1/3 of pre-commit votes. Validators are selected based on the stake held in their wallets, and the stakes are locked with the digital signature of the validator, permitting the network to revoke the stake as a penalty in case of malicious behavior.

##### $IoT\_Adaptability\overset{}{\to }Not\_IoT\_friendly$

Tendermint consensus is very scalable and secure and can be implemented in IoT applications. However, the monetary concept related to the PoS part is unsuitable for the IoT systems.

##### ByzCoin [84]

ByzCoin is a combination of PBFT and PoW consensus algorithms. ByzCoin uses a specialized communication protocol based on a tree structure, helping to significantly improve the latency of the network. Furthermore, ByzCoin consensus depends on the Cosi (collective signing) protocol, which helps to sign a new transaction in seconds. Cosi and tree-structured communication protocols significantly improve transaction throughput. However, ByzCoin is not secure enough, particularly against DDoS attacks.

##### $IoT\_Adaptability\overset{}{\to }Not\_IoT\_friendly$

Although ByzCoin has high throughput, its security limitations, and PoW dependability make it the least favorable for IoT applications.

##### Algorand [85]

Algorand is built on pure PoS and BFT (BA). The focus of this algorithm is security, decentralization, and scalability. Decentralization is achieved by randomly selecting the committee to generate new blocks using a non-interactive protocol based on the private key and public information of each member, and the stakes associated with their accounts (pure PoS). The security and scalability of this algorithm are achieved using the BA protocol, which is used to finalize the block proposed by the committee (Byzantine agreement). Classic BFT algorithms are vulnerable to the Sybil attack. However, BA removes this threat with the help of the random selection of committees in the first step.

##### $IoT\_Adaptability\overset{}{\to }Not\_IoT\_friendly$

Algorand is built on Pure PoS, and the committee selection is based on the staked value in the network. This monetary concept makes the Algorand consensus not suitable for IoT applications. PoA, which is already discussed in side_chains, can also be classified as a collaborative consensus as it uses both PoS and PoW, although PoA was particularly proposed for Bitcoin. Therefore, it is listed under side_chains.

**Figure 8:**
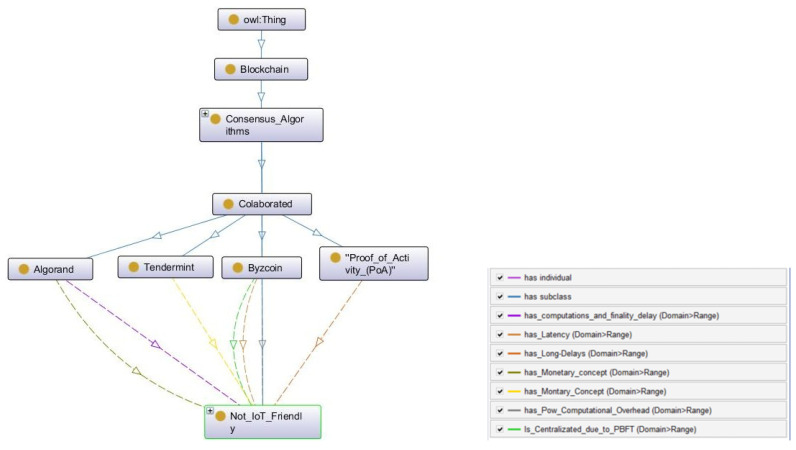
Ontology of collaborative consensus class algorithms and IoT adaptability.

Collaborative consensus methods have the ability to increase the performance of any consensus algorithm. In terms of the fit-to-use case, the combination of multiple consensus methods can be good for IoT_Adaptability. However, no collaborative consensus algorithm has been proposed for the IoT environment. Furthermore, it requires careful consideration when combining the algorithms by eliminating the limitations of original algorithms. Collaborative ontology is presented in Figure 8.

From the detailed discussion of consensus algorithms in CONB, it is clear that most of the blockchain-based systems are proposed for financial applications. Such consensus algorithms are designed for high computational power (competitive), high stakes (comparative), centralized authorities within the democratic decision (vote-based), overlays for financial blockchains (non-linear), and use case (not IoT)-based collaboration of different consensus functions (Collaborative). Such properties are not favorable for the IoT environment with resource-restricted sensors and devices. Moreover, the intentional delays to ensure the security of the network in these algorithms would pose another hindrance to time-critical IoT systems. A few algorithms, such as SCP, ripple, or PBFT, can be considered for IoT applications if some factors, such as small/close networks, low fault tolerance, or tad system overheads, can be ignored. Furthermore, from the IoT adaptability description of every consensus algorithm (except competitive), every consensus algorithm has the potential to be used for multiple use cases, including IoT, with few optimized parameters. On the other hand, there are some consensus algorithms, which are particularly proposed, designed, and implemented to work with the IoT resource-limited environment. These algorithms belong to non-linear and comparative classes in the CONB ontology and are discussed in detail in the following CONIoT ontology.

### 3.2. CONIoT

CONIoT consists of consensus algorithms, which are formulated for IoT applications. *Blockchain_for_IoT* is a main class in the *Owl:Thing* hierarchy and has two subclasses, *IoT_Friendly_consensus_algorithms* and *IoT_Applications*. First, *IoT_Friendly_consensus_algorithms* have two more subclasses: (1) DAG: This is an extension of the non-linear class, and (2) Proof of *: This is an extension of the comparative class of CONB ontology. DAG consists of nonlinear data structures and block finality methods, contradicting the blockchain data structure, while Po* consists of optimized versions of comparative consensus. *IoT_Applications* consist of the associated implementation details or possible outcome of the proposed consensus mechanism. Although these algorithms are implemented in IoT environments, they still have some limitations and trade-offs, which are represented as ObjectProperties and are linked to *IoT_Applications* using domain > range. Furthermore, the definitions of concepts are added as annotations under rds:comments.

#### 3.2.1. DAG

**Figure 9:**
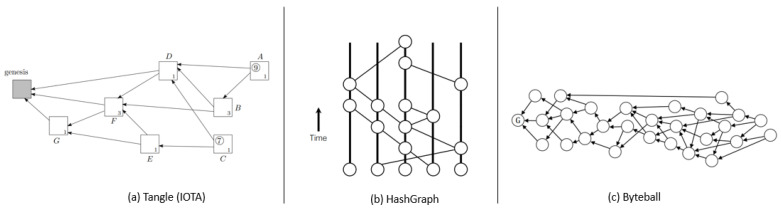
Comparison of direct acyclic graph-based blockchains.

The DAG-based blockchain is considered an alternative to the traditional blockchain due to its parallel transaction confirmation rather than building linear blocks in a blockchain ledger. However, the underlying techniques, such as transaction confirmation, ledger finality, cryptography rules, and decentralization are inherited from traditional blockchains. DAG-based systems are still under development compared to other blockchains due to the contradictory design (nonlinear), limited standards (variate from one DAG blockchain to another), diverse execution as represented in Figure 9, and the limited implementation or applications. Furthermore, DAG-based systems are not formalized uniformly. Although they do not cover all key points (rules and defined parameters), they still show a promising future through the modification of DAG for mainstream applications. On the other hand, the scalability and performance of the DAG-based blockchain have drastically improved by eliminating the waiting time, computation, stakes, and overlays, rendering it well-fitted for IoT systems. There are many other DAG-based systems, such as Nano [86], Graphchain [87], Blockmania [88], etc., which are developed using DAG. However, they are not added as subclasses of DAG because either these works are not explained well or do not fall under IoT adaptability. In CONIoT, the DAG class has three sub-classes, IOTA, Hashgraph, and Byteball. These projects use the DAG data structure but use entirely different mechanisms for transaction validation, finality, and incentives. The vertices and edge structure of these blockchains are presented in Figure 9.

##### IOTA (Tangle) [89]

IOTA is a project designed to work with IoT systems, considering resource scarcity, and it uses Tangle as a consensus algorithm. Tangle is a very optimized consensus algorithm, which is based on the DAG. In Tangle, every new transaction must confirm or validate two randomly selected previous transactions. Tangle carries out directed random selection based on the Markov Chain Monte Carlo (MCMC) to avoid lazy tips by assigning a weight to every transaction (self-weight and cumulative weight). These weights depict how many times a transaction has been verified by new transactions and how many times the new transaction is verified by latter transactions. Based on this weight, the strongest Tangle can be verified. Furthermore, Tangle is not a pure block structure; hence, a transaction does not have to wait to be mined. Consequently, it is very scalable, which is good for the IoT systems. However, there is some system overhead for the Tangle-based system, as Tangle uses the ternary system. The interoperability of IoT requires power consumption. Moreover, the initial version of Tangle had coordinators to ensure the integrity and completeness of the system, which tends toward centralization. Recently, in 2020, IOTA launched the Coordicide Alphanet, a coordinator-free network, a smart contract layer, transaction rate control, etc. Moreover, the Tangle node uses the PoW. This is unlike Bitcoin or Ethereum, which use SHA-256 to reach a consensus. In Tangle, the PoW is used to avoid spamming and its main purpose is only to find a nonce for a transaction to be part of the Tangle ledger by using the SHA-1. However, IoT devices are not usually powerful enough to do this kind of computation; thus, the computational work is usually offloaded to the proxy servers. IOTA uses PoWbox as a proxy server, and IoT devices only need to generate the system call through the UI without installing or configuring on local devices. AWS lambda is also compatible with Tangle. Tangle is built with the intention of scalable transactions. By design, there is no miner and everyone making a transaction confirms that the previous transactions are part of the ledger. Upcoming transactions confirm those previous transactions in a parallel fashion, and so on. Hereby, every user is a miner. Every transaction is considered a node, and new transactions are linked in the unidirectional ledger. The main characteristics of Tangle are feeless transactions (microtransactions), quantum resistance, and Low_latency, which are listed as ObjectProperties. These ObjectProperties are linked with the General_DAPPs (general IoT DAPPs) using the domain > range description in the CONIoT Ontology. Furthermore, literature studies that are based on the Tangle consensus are listed as *instances* of the subclass IOTA, such as distributed communication protocol [90], Streamnet [91], and credit-based consensus [46].

##### $Consensus\_for\_IoT\overset{}{\to }Applications$

IOTA by design is very scalable, unlike other blockchains, the scalability of the system increases as the number of transactions increases. feeless structures and scalability make IOTA very desirable for IoT applications, such as smart charging [92]. In this work, the authors compared the performances of Ethereum and IOTA. It concluded that IOTA is more appropriate for this kind of application, i.e., IOTA does require specialized software (such as EVM) to run the smart contracts, and there are no confirmation delays (PoW (Ethereum 1) or PoS (Ethereum 2)), and users can perform microtransactions on runtime. Furthermore, IOTA applications are not limited to microtransactions [93] but are applicable to access control [94], fairness of the distributed ledger [95] (IoT), security (DDoS prevention) [96], etc. IOTA is new and needs a lot of research and practical implementation models. Therefore, in CONIoT the ObjectProperties link of IOTA is made to the General DAPPS using domain > range descriptions.

##### Byteball [97]

Byteball is a decentralized arbitrary data storage solution built on the DAG model. Byteball does not work as a normal database; however, it stores the data with associated values, such as stakes, currency (Bytes), property titles, etc. The same with the legacy blockchain, in Byteball, data are cryptographically linked, and new transactions store hashes of previous transactions to keep a partial order. Byteball stores data without central involvement. The new transaction pays the network fee according to the data size, and that fee is paid to upcoming transactions as a confirmation reward. As the number of new transactions increases, the ledge becomes more secure by obtaining its hashes stored in multiple new transactions either directly or indirectly. The Byteball consensus mostly works as Tangle because all confirmed transactions must be directly or indirectly verified by the tips. However, the main difference is the transaction confirmation procedure. In Byteball, transactions are confirmed by the 12 reputable witnesses, which are specific types of users trusted by the nodes for confirmation and are used to avoid the double spending problem. Moreover, these witnesses are assumed to be honest and vote for the position, which makes Byteball somewhat centralized. However, optimized consensus and storage can be well-suited for IoT setting.

##### $Consensus\_for\_IoT\overset{}{\to }Applications$

Byteball architecture is ideal for IoT applications, and the DAG implementation provides significant scalability and performance efficiency. The technical setup of the Byteball ledger is very straightforward for IoT systems. The Byteball core can be set up on any NodeJS-supporting node and it starts working within seconds. Moreover, the Byteball Core does not require any extended hardware and can work on 128 MB RAM and 8 MB for data storage. These minimum system requirements can be implemented in many IoT applications, using the Byteball network to store the data from IoT sensors or even micropayments. Unlike IOTA, which is entirely feeless, Byteball has associated fees. However, these fees are based on the transaction sizes not fixed, as in the mainstream blockchain Ethereum or Bitcoin, where micropayments are impossible because network fees are higher than the actual transactions in most cases (micropayments). In CONIoT, the ObjectProperties of Byteball are linked with General DAPPs using domain > range descriptions. Furthermore, BIoT is an *instance* of the Byteball subclass, BIoT is a project proposal for micropayment solutions for IoT networks [98].

##### Hashgraph [99]

Hashgraph is another DAG-based solution. The consensus of Hashgraph is similar to Byteball and Tangle, but the transaction verification procedure is more complex. Verification does not only depend on tips (Tangle), or witnesses (Byteball). Nonetheless, it must be verified by 2/3 (Byzantine agreement) of the nodes, multiple times. This extra measure is to avoid fork attacks. Moreover, the blocks known as events are generated by the nodes and must be two-parent events, implicating self-parent and being generated by the neighbor node. The process is the same as the tips that verify two previous transactions, but the verification measures are different in Hashgraph. Furthermore, the Hashgraph voting model is based on virtual voting. We calculate the vote of neighboring events based on the information shared by those neighboring nodes. This approach significantly reduces the system communication overhead in the decision-making process. Hashgraph inherits the scalability and performance efficiency of the DAG architecture. Hashgraph is built on the low and fixed-fees model, which makes it ideal for micropayments as in other DAG-based blockchains. Furthermore, Hashgraph is dedicated to working on the IoT adaptability issue. A project named ’Trusted IoT Alliance’ is open-source software that helps to take advantage of the features of blockchain and IoT intersections. The aim is to work on IoT applications to provide digital/cryptographic identities of devices. Supply chain sensors (RFD, QRs), vehicles of the supply chain, etc., are controlled by smart contracts. Multiple proofs of concept and testbeds are published as open-source standards under this alliance.

##### $Consensus\_for\_IoT\overset{}{\to }Applications$

**Figure 10:**
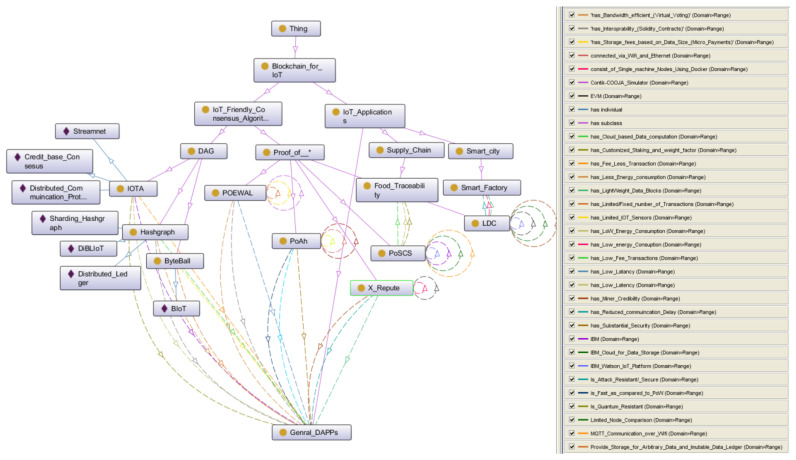
Consensus ontology for IoT.

Hashgraph ObjectProperties, such as bandwidth_efficiency (Virtual voting), interoperability, and low-fee transactions are malleable for IoT applications. These ObjectProperties are linked with general DAPPs using domain > range descriptions in the CONIoT. Furthermore, literature using Hashgraph architecture in multiple IoT examples are listed as instances of the Hashgraph subclass. These properties of Hashgraph are used in many IoT-based applications. In [100], the authors used the Hashgraph consensus algorithm for the classification of IoT devices using Hedra [101] (the Hashgraph consensus as a cloud service). In [102], it presented a high-performance decentralized architecture for many IIoT use cases. It introduced a node evaluation system using the sharding Hashgraph consensus mechanism based on node evaluation for a large number of devices connected into shards. Furthermore, in [103], it presented a comprehensive study of Hashgraph consensus and presented its efficiency compared to HoneyBedger [104] and BEAT [105]. It concluded that Hashgraph can be used on many mainstream applications because of its low latency, high throughput, and low communication overhead. DAG-based blockchains promise fast transaction confirmation and scalability by adding transactions parallel to the ledger. Due to the unique topology of DAG being without direct cycles, it provides a very scalable data structure for blockchain-based IoT applications. The CONIoT owlGred view is presented in Figure 10.

##### DAG-Based Blockchain Comparison

It is evident from the above discussion on the DAG class and its subclasses that the implementation of every DAG-based blockchain is entirely different. All three selected DAG-based blockchains used different criteria for transaction validations, finality, fees, etc., which consequently have different use cases and applications. A detailed comparison of IOTA, Hashgraph, and Byteball is presented in Table 2. Furthermore, the comparison was divided with respect to the transaction validation process, degree of centralization, monetary concept, security, DAG, IoT, and generic characteristics. The common objective of the DAG-based blockchain preferred for this study is ‘scalability’, which is the most important feature of DAG compared to the legacy blockchain when it comes to IoT adaptability. Further comparison criteria of IOTA, Hashgraph, and Byteball are discussed below.

##### Transaction Validation

The transaction validation process can be defined as a process of how a transaction becomes part of a decentralized ledger and a process to ensure the integrity of the transaction. IOTA, Hashgraph, and Byteball have the same criteria (based on vertices) but differ in the implementation aspect. Every new transaction is handled as a node and multiple transactions can be validated in a parallel fashion, making these blockchains exponentially scalable as compared to the legacy blockchain, such as bitcoin or Ethereum. The new transactions act as validators of previous transactions and are cryptographically linked in a parent–child relationship (but not linear blocks). The number of previous transaction confirmations differs. In IOTA, a new transaction is bound to validate two previous transactions, and the previous transactions are selected based on the MCMC algorithm, which depends on random but recent criteria. On the other hand, Hashgraph and Byteball do not have defined numbers of previous vertices. Instead, they select random vertices, such as neighbors (Hashgraph) and not matching parents (Byteball). Consequently, this transaction validation process removes the need for miners in the network either based on computational power, stake, or voting, which are basic hindrances of IoT adaptability of blockchain, as discussed in CONB. Furthermore, the global state and finality of these blockchains are significantly improved. Rather than waiting for the longest chain, the finality is achieved on the weighted threshold and the maximum witness level, which noticeably reduces the confirmation time. Therefore, DAG-based transactions are well-suited for IoT applications. However, these transaction validation methods are only scalable when the number of transactions or data propagation of the network is high. If there are no upcoming transactions, then tips (or, lastly, added transactions) are dependent on the coordinator or centralized authority validation.

##### Degree of Decentralization

The DAG-based blockchains are designed as a public decentralized ledger, which has completely removed the governance issue of the first- and second-generation blockchains. As in the first- and second-generation blockchain, the miners or validators consist of separate networks rather than actual users who make transactions. This type of blockchain governance gap is unacceptable for the IoT application. The DAG-based blockchains completely remove the concept of miners and validators, but the actual transaction acts as the validator by giving equal importance to every network participant. However, these blockchains use legacy consensus algorithms. For example, Tangle uses PoW as the anti-spamming method to restrict every upcoming transaction (to wait for a specific time period). On the other hand, these blockchains are somewhat centralized in nature when it comes to the current ledger state.

##### Monetary Concept

As we have discussed in the class of CONB ontology comparative consensus methods, the monetary concept of these consensus methods is the reason why they cannot be implemented in IoT applications. The IoT systems require feeless/low-fee structures where transactions involve monetary value transfers as well as data (sensor readings). In these chains, there are either no fees (Tangle) or very low fees, depending on the transaction (Hashgraph) and data size (Byteball).

##### Security

DAG-based blockchains are also prone to security threats, such as forking, double spending, and malicious nodes. As the data structure of the DAG-based blockchain is entirely different, so are the security measures. There are no significant differences between the performances of these security measures as compared to legacy blockchains. It is all about the implementation details. Forking, fault tolerance, and double spending issues are tackled as interlinked techniques i.e., cumulative weight (IOTA), strongly seeing (Hashgraph), and best parent (Byteball). Moreover, the immutability of DAG-based blockchains is also dependent on cryptographically linked data, which impose encryption/decryption overhead on the system. However, this system overhead is drastically less than the block-structured blockchain where the hashes are built on the nonce, multiple transactions, and previous block hash, which are required to meet the target difficulty.

##### DAG and IoT

DAG-based IoT applications are discussed in the CONIoT class DAG. The key elements of DAG-based blockchain and IoT adaptability are the least hardware-dependent. IOTA, Hashgraph, and Byteball do not require any specialized hardware to run their instances, which makes them ideal for IoT applications. DAG-based blockchains are well-suited for microtransactions used in electric vehicle charging stations, and decentralized ledgers. The specific application and implementations of DAG and IoT are presented as instances of DAG subclasses in CONIoT.

##### General Characteristics

The overall non-linear data structures, consensus methods, finality, and security measures of DAG-based blockchains are ideal for IoT applications. DAG throughput has no upper bound in that the throughput increases as the number of transactions increases in the network. The distinctive features of IOTA, Hashgraph, and Byteball, e.g., fairness of order (Hashgraph), are very important for the IoT application as compared to the first and the second-generation blockchains where transactions are selected for the next block based on the associated fee (transaction with high fees are preferred) instead of the order of time-stamping. However, there are limitations in DAG-based blockchains. The most important one is centralization. Moreover, these solutions are not well-tested.

A DAG-based data structure not only improves scalability due to its non-linear data structure but also removes the high computational strain from IoT systems. However, these methods are comparatively new and more efforts are needed to make them work in real-life IoT applications.

#### 3.2.2. Proof of * (Po*)

Po* is a subclass of *IoT_Friendly_consensus_algorithms* and consists of consensus algorithms, which are specifically proposed for IoT applications. Each algorithm is presented as a solution to a particular use case that is listed under the application subclass of *Blockchain_for_IoT* in CONIoT. These applications are linked with ObjectProperties using domain > range descriptions. Furthermore, these algorithms are presented as academic contributions and, thus, have implementation details as well. These details are added as Experimental_Evaluation class under *Owl:topObjectProperty* hierarchy. Moreover, relevant details about these classes are added as rds:comments using annotations. These algorithms are defined as Proof of * where * is the standard used for the consensus. These algorithms belong to the comparative consensus in CONB ontology.

##### Lightweight Data Consensus (LDC) [106]

LDC has been proposed as a lightweight consensus mechanism for Industrial Internet of things (IIoT) for secure data transmission in many smart city use cases. It stores a distributed ledger on edge gateways in lightweight blocks. For data consistency, it uses a two-path routing strategy. This work helps to solve the traditional consensus and data structure issues, e.g., high storage requirement, which is tackled by using lightweight blocks and edge gateways, acting as less resource-scarce units compared to IoT devices. LDC is a subclass of Po* in the CONIoT ontology. It has ObjectProperties listed under Owl:topObjectProperty hierarchy. These properties are linked with the IoT_Applications’ subclass of *Blockchain_for_IoT* on the *Owl:Thing* hierarchy using domain > range descriptions. Furthermore, the Experimental_Evaluation of LDC is also listed under the *Owl:topObjectProperty* hierarchy as Limited_node_comparison.

##### $LDC\overset{}{\to }Application\;and\;Experimental\;Evaluation$

The authors state that in most IIoT systems there is a single edge gateway that poses a single point of failure issue and the physical latency of that gateway affects the communication delay. They presented a Smart_factory solution with Limited_node_comparison by using a virtual multi-grid gateway in a sensor network. Sensor data are collected by the nearest gateway (auctioneer) and this gateway forwards the hashed value of that data to two adjacent edge gateways (bidders). Communication between gateways is handled in the same way as a game model of data forwarding until the destination edge gateway. Destination edge gateways build data blocks and share them with verification edge gateways, which further verify the data ledger state using consensus and send the data to a data center. Furthermore, they evaluated their work by comparing it with PoW, single node (single gateway), and multi-edge gateways. According to the results, LDC significantly reduces communication delay, improves data consistency, and has the least energy consumption.

##### $LDC\overset{}{\to }Consensus$

The LDC consensus is achieved at the verification and destination edges. Destination edges receive data and propagate them to multiple verification edges. Verification edges verify the data by comparing it with previous ledger hashes. If the validated data receive more than 50% of the votes, then they are considered as the final ledger state and are sent to the data center. LDC provides a lightweight block structure, low energy consumption, and reduced communication delays. However, implementation of the proposed consensus method has only been tested on limited nodes. Moreover, the multi-edge gateways themselves put an extra system overhead in an IoT system. This system can work in the IIoT where multi-edge devices can be installed as part of the sensor network. However, it cannot be taken as a universal consensus for IoT adaption.

##### Proof of X-Repute (X-Repute) [107]

X-Repute builds on the PoX consensus protocol, and the contribution of this work is to add the reputation of every node in the mining process where the focus is to increase the security and credibility of the process. It targets the reputation of every node by Miner_Credibility_Check, introducing the reward and punishment method. Furthermore, the credibility check is performed before starting the mining process. Therefore, malicious nodes are determined before starting the mining; thus, no malicious nodes in the mining process. Moreover, it determined the malicious nodes based on the network delay. If a particular threshold is achieved, then the node is doomed as malicious and cannot participate in the mining process. XRepute ObjectProperties Miner_credibility, attack_resistant/secure, and Low_energy_consumption are listed under *Owl:topObjectProperty* and are linked with General_DAPPs using domain > range descriptions. The Experimental_Evaluation of X-Repute is listed as EVM in CONIoT.

##### $XRepute\overset{}{\to }Application\;and\;Experimental\;Evaluation$

In this study, experimental evaluation was not presented for a particular use case. Hence, it was linked to the General_DAPPs. Furthermore, it experimentally evaluated the proposed solution on an Ethereum virtual machine (EVM) on a single machine node using Docker.

##### $XRepute\overset{}{\to }Consensus$

X-Repute consensus inherits the properties of the PoX consensus, which is built on stake and collateral. In the credibility check, a miner will build its reputation on either if it is good or bad and will be rewarded or punished accordingly as collateral. This method is built for IoT systems to avoid the forks and double spending issues in later stages. It introduces the miner’s credibility at the very beginning of the process. X-Repute is secure due to the credibility check. Due to PoX properties, it works on stakes and collateral. Subsequently, it does not require high computational power. Nevertheless, the experimental results are conducted on Ethereum, and nodes are set up on a single machine using Docker. Therefore, this implementation has two limitations. First, if the setup is running on the Ethereum blockchain, then it means, by default, it is inheriting the PoW consensus. Second, the nodes are on a single machine, so the network delay is neglected.

##### Probabilistic Proof of Elapsed Work and Luck (PoEWAL) [108]

PoEWAL is proposed for non-cooperative IoT systems to improve the robustness and reliability of every IoT device participating in the consensus method. PoEWAL is designed as a set of clusters. Every cluster consists of resource-constrained devices, such as sensors, and a cluster head that has enough resources to receive data from sensors and does node-based computations. All clusters receive data from IoT devices in the corresponding cluster and send the data to the base station. PoEWAL is listed as a subclass of Po* in the CONIoT, and has ObjectProperties Low_latency, less_computational_power, and Low_energy_consumption. The Experimental_Evaluation is listed as Contik_COOJA_Simulator in the *Owl:topObjectProperty* hierarchy. Furthermore, the PoEWAL is linked with General_DAPPs in the application subclass using domain > range descriptions.

##### $PoEWAL\overset{}{\to }Application\;and\;Experimental\;Evaluation$

For the experimental results, the authors used Contik_COOJA_Simulator with limited sensors that could be used for temperature, light, or humidity sensors. They divided the implementation into four phases. Phase 1: it solely focused on the PoEWAL performance in terms of latency, computations, and energy consumption. In Phase 2, it compared the experimental results for different consensus methods, such as the PoW, PoS, and PoA in terms of the computation cost. Furthermore, phase 3 focused on the energy consumption comparison of the selected algorithms, while in phase 4, it compared the latency experimental performances.

##### $PoEWAL\overset{}{\to }Consensus$

The consensus was based on solving a puzzle not as difficult as in the PoW. All cluster nodes mine new blocks in finite time, and the node with the highest number of consecutive zeros can mine the block. If a collision happens, then proof of luck is implemented in a way that the miner with the lowest nonce will be selected as the winner. PoEWAL uses an improved difficulty level of PoW, which significantly reduces the computational power as presented in the Experimental_Evaluation. Furthermore, according to Phase 1 of the experimental setup, the energy consumption is reduced. However, few implementation details, such as communication between cluster nodes and base station, and how the base station maintains the data on the blockchain, are missing.

##### Proof of Authentication (PoAh) [109]

PoAh by design is developed for transaction authentication instead of validation, in contrast to the other consensus algorithms, such as the PoW, PoS, etc. Furthermore, PoAh can only be implemented in a permissioned or private network as it depends on trusted nodes. Only trusted nodes are responsible for creating new blocks, and the rest of the nodes only update their ledgers with a simple check (whether the block is propagated by a trusted node or not). Due to authentication instead of validation, PoAh requires minimal resources as the mining process of PoAh uses a digital signature for block validation. Furthermore, the PoAh has a very low latency as compared to the PoW (10 min). This is because it utilizes the digital signature for cryptographic authentication, which takes significantly less time than competitive puzzle-solving. Moreover, PoAh is substantially secure and has eradicated the 51% attack by selecting trusted nodes. PoAh is also listed under the Po* Class; the ObjectProperties of PoAh, e.g., fast_as_compare_to_PoW, substantial_security, and utilize_minimal_resources are listed under the *Owl:topObjectProperty* hierarchy. These ObjectProperties are linked with the General_DAPPs subclass of Application. The Experimental_Evaluation is linked with the subclass NodeRED using domain > range descriptions.

##### $PoAh\overset{}{\to }Application\;and\;Experimental\;Evaluation$

The PoAh experimental evaluation is based on two methods. First, it is based on the python-based simulation with a defined number of trusted nodes, a 35-byte block size, and ELGamal cryptography. As this system consists of every sensor, the block formation consists of the MAC address, signature, and transactions. Second, the authors presented another simulation with real-time sensors consisting of Raspberry Pi single-board computers. Multiple sensors collected environmental data with slightly different processing power. They also installed the trusted nodes in the network. Furthermore, for the PoAh consensus algorithm, they used the NodeRED tool. The experimental results show that PoAh is better than PoW in terms of latency, computational power, and energy consumption.

##### $PoAh\overset{}{\to }Consensus$

Every device on the network generates a block with its digital signature and presents it to the trusted node. Then, the trusted node uses the public key to validate blocks, and authenticated blocks are broadcast to the chain. Although PoAh promises very good characteristics, which are essential for IoT networks, IoT networks require more scalable and well-defined consensus mechanisms. Due to requirements on the number of trusted nodes in PoAh, it is a centralized system (to a certain extent) and the blockchain puts a storage overhead on the system consisting of resource-constrained IoT devices. Moreover, the assumption that the trusted node can never be malicious is not practical in a decentralized system, and some fault tolerance techniques will be needed.

##### Proof of Supply Chain Share (PoSCS) [31]

PoSCS is proposed for a supply chain application with food traceability using lightweight blocks, and the lightweight consensus is mapped on a cloud-based infrastructure. It proposes an optimized consensus mechanism as compared to legacy blockchain consensus methods, which cannot be implemented in an IoT environment. Furthermore, the proposed consensus method consists of a shipment transit time, shipment volume, and stakeholder assessment. Moreover, it proposes a blockchain hybrid approach: IoT sensors provide data to the application layer using MQTT over the Wi-Fi network, and data are stored and processed over the Cloud. PoSCS is the last Po* consensus method in the CONIoT. The ObjectProperties include cloud_based_data_computations, customized_staking_and_weight_factor, and Low_energy_consumption under the Owl:topObjectProperty hierarchy. The ObjectProperties of PoSCS are linked with Food_traceability, which is a subclass of the supply chain Application in the *Owl:Thing* hierarchy of CONIoT. Lastly, the Experimental_Evaluation is represented under the IBM subclass.

##### $PoSCS\overset{}{\to }Application\;and\;Experimental\;Evaluation$

To evaluate this work, the article implemented the proposed solution in an e-commerce store using IBM cloud as data storage, IBM Watson IoT platform, and MQTT communication over Wi-Fi. It divided the implementation into four phases: (1) Phase 1. It implanted the Watson IoT system to collect sensor data; (2) Phase 2. It established an integrated blockchain engine with consensus mechanisms; (3) Phase 3. Fuzzy logic implementation was conducted for shelf life monitoring and adjustment; and (4) Phase 4. It proposed a DAPP for the traceability of e-commerce data.

##### $PoSCS\overset{}{\to }Consensus$

The consensus method is similar to the PoS where validators of the blocks are stakeholders. As validators are actual stakeholders, there are four factors to decide who will mine the next block. Each factor contains a weight; based on the collective weight, the validator block is selected and considered the final block. The weight factors include SAT, DEV, INF, and INT (α range (0,1)). In addition, the transit time and shipment volume are considered in the validator selection.

It is evident from the Po* discussion, the consensus mechanism listed in this class is limited to particular use cases. In comparison with legacy consensus methods, such as PoA, PoS, or PoW, the performances of these algorithms significantly improved. However, the paper did not compare its work with more scalable solutions, such as DAG.

The consensus algorithms discussed in the CONIoT are especially proposed, considering the IoT environment and its resource limitation. These algorithms significantly improve the scalability and computational issues. However, these algorithms are use-case dependent and do not provide a standard solution.

## 4. Challenges and Future Directions

The distinctive features of blockchain, such as immutability, transparency, autonomy, and anonymity are useful for many IoT-integrated systems, e.g., smart cities [2,3,9,12,110], e-healthcare [33,34,35], smart factories [24,25], and electronic vehicles [111], etc. However, IoT-based systems consist of different heterogeneous low-end devices, i.e., actuators, sensors, and smart communication devices. Furthermore, these resource-constrained devices are vulnerable to security attacks and require lightweight algorithms (encryption/decryption). The main challenge of labeling IoT adaptability is its dependence on a specific problem. Every use case sets a distinct requirement and needs customized solutions. However, we can identify the most common challenges for the IoT system as follows. Computational power: Current blockchain protocols typically depend on high computations for puzzle-solving or cryptic data processing, putting a strain on resource-constrained IoT environments. Storage: In a blockchain, to reach a decision, every participant in the consensus mechanism validates the proposed block by comparing the hashes of the previous ledger, which requires data replication at each node. Though the concept of the light nodes can address this problem to some extent, some IoT environments do not have such storage capacity. Data privacy and security: In most IoT environments, data transmission of IoT devices is deemed private. Therefore, privacy preservation of communication is compulsory. In resource-constrained IoT environments, how to avoid multiple layers of software-based privacy (due to resource limitations) while still preserving privacy is a big challenge. Furthermore, current blockchain security is dependent on advanced cryptographic calculations, which are resource-demanding. Scalability: IoT systems often consist of a large number of sensors and/or small devices and need to be connected concurrently. Therefore, IoT networks must be scalable in terms of data transfer capacity or reaching consensus. Furthermore, IoT applications are usually time-critical and built upon exponential and synchronized communication links; hence, network latency must be insignificant.

The above-mentioned challenges highlight various research gaps in the current state of blockchain with respect to IoT adaptability. They provide research directions toward eventual implementation of blockchain technology in real-life IoT environments. The current consensus algorithms [69,70,75] (even the nonlinear solution [88,96,98]) depend on PoW, which is comparatively expensive and Not_IoT_friendly. Although some improvements have been made in addressing these issues, such as PoS algorithms, which do not depend on computations but stakes, it will still be a long way before they can be implemented in IoT systems due to their strong dependency on monetary concepts. Therefore, it is very important to define a new standard for block creators and validators, which does not depend on these expensive (computationally or monetary) concepts. Another challenge is to find how data are optimally stored for blockchains in IoT environments. Current consensus focuses on the validation of new data in a ledger based on the previous state of the ledger, putting a storage capacity strain on the IoT environment. Another major research direction is how to optimize the validation process and define new parameters for the finality of the current state. Lastly, the scalability of blockchain software, given the underlying network limitations is crucial for IoT environments. DAG-based solutions particularly focus on the scalability issue for IoT environments. They show that applying new data structures could be an effective way to address many of the current scalability issues for IoT applications.

## 5. Conclusions

The decentralized nature of blockchain technology is considered very ideal for autonomous IoT environments. However, due to high computational requirements, existing consensus algorithms are not feasible in the resource-constrained IoT setting. Although the Po* and DAG-based consensus algorithms are IoT-friendly, they are not yet standardized for mainstream IoT adaption. In this study, we introduced a new way of understanding and classifying blockchain consensus algorithms with regard to their applicability to IoT use cases. We also presented a formally specified ontology for blockchain consensus algorithms, allowing reasoning about the properties of the algorithm’s ontology. We demonstrated the use of ontology by applying it to the vast literature on blockchain consensus algorithms, which enabled us to understand their limitations with respect to the IoT application. Furthermore, our work provided a formalized review of existing works and pointed out the gaps and directions for future research.

## Figures and Tables

**Table 1:** Related Surveys on Consensus.

Survey	Consensus Algorithms					Scalability Analysis		DAG			IoT-Adaptability
	PoW	PoS	BFT	Po*	OTGCA	Latency	Throughput	Stated	ID	CA	
Bouraga [54]	✓	✓	✓	✓	X	✓	✓	✓	X	X	X
Bodke et al. [55]	✓	✓	✓	✓	RAFT, PAXOS	✓	✓	✓	X	X	X
Salmitari et al. [56]	✓	✓	✓	✓	Ripple, Stellar, Raft, Elastico	✓	✓	✓	X	X	X
Fu et al. [57]	✓	✓	✓	✓	Algorand, Tendermint	✓	✓	✓	X	X	X
Lao et al. [58]	✓	✓	✓	✓	Ripple	✓	✓	✓	X	X	X
Fernandez et al. [59]	✓	✓	✓	✓	X	✓	X	X	X	X	X
Our Work	✓	✓	✓	✓	✓	✓	✓	✓	✓	✓	✓

Po*: Proof of (time, reputation, identity, etc.); OTGCA: other third-generation consensus Algorithms; ID: implementation details, CA: critical analysis.

**Table 2:** DAG-based blockchain comparison.

Characteristics	Tangle [89]	Hashgraph [99]	Byteball [97]
Objective	Microtransaction solutions for resource-constrained systems and scalable decentralized ledger	Distributed ledger for microservices with low-fee transactions and scalability	Tamper-proof storage of arbitrary data
**Transaction Validation**			
New transaction handling	Validate two previous transactions based on strong Tangle (cumulative weight-based) and conduct a basic PoW	Payload new transactions are added on the vertices and gossiped to the other vertices (events) with a timestamp and self-ancestor	New transactions (units) are added by 1 of 12 witnesses and BEST PARENT is selected to link with the MainChain. Use SKIPLIST is not sequential or linear
Maximum previous vertices selection (previous transactions)	Two	Random	Random
Confirmation Delay	Depends on a new transaction arrival time	Not defined	Ideally 30 s, intended
Criteria to select previous vertices	Random walk, MCMC	Random gossip protocol	If x reference y then z cannot reference both x and y
Finality	As soon as the cumulative weight reaches the confirmation threshold	Famous Witness. A random Hashgraph vertices are selected and calculated by the BFT protocol for a fair total order of the graph	If max_wl min_wl of the branch is in question, then it is doomed invalid, otherwise the finality is achieved
Global state	Global state is currently dependent on the coordinators; however, the strongest Tangle to the Genesis block is considered the Honest Tangle	Gossip about Gossip History. All participants have a history of the ledger and the strongest history by 2n/3 votes is the global state	MainChain (MC). Starting from different tips and reaching to intersection vertices, forming a link until the Genesis block
**Degree of Decentralization**			
Decentralization	Coordinate-dependent	Enterprise level solution	Consortium, witness-dependent
Use of legacy consensus protocol	PoW (not the same as other blockchains, just to calculate nonce)	BFT	None
Consensus	Coordinator-based; a timely intervention by coordinators to verify a Tangle validation	Hashgraph consensus. The consensus is met by a virtual voting process and is agreed upon using the BFT YES/NO agreement on the current ledger state	Witness-Based MC with more witnesses; every witness is counted once (reality test)
**Monetary Concept**			
Transaction Fees	NO	No fees to make the transaction on the network but micropayment network fees apply	Yes
Fee charging criteria	None	Network fees	Fee is charged (new tips) according to the data size on the ledger
Incentives	None by the design. Layer two implementation will have incentives, depending on the global state consensus protocol	Fixed network fees	Fee charged according to the data size is an incentive to data validators (New Tips)
Native currency	MIoTA	Hedera	Bytes
**Security**			
Double spend	Yes	Yes	Yes
Double spend handling	The combination of validation time, PoW nonce calculation (intended delay), MCMC random tip section and timestamp used to avoid double spending and the transaction with a low CW ‘domed’ invalid	The same as fork handling; only deep in the chain transaction is considered	All transactions from a single user must be in serial, otherwise, it is considered a double spend; the first transaction will be considered valid
Forking	YES. Parasite chain	YES. Conflicting gossip to random vertices	Yes. Shadow DAG
Fork resolution method	Forking in the Tangle is handled as a double spending attack, considering the unfair entity makes many microtransactions to validate the malicious transaction, increases the depth of that transaction and they are handled the same as a double spending attack	If ancestor and self-ancestor do not have the history (gossip about gossip) about strongly seeing gossip, then that transaction and the transaction attached (Gossiped) to that sequence are considered ‘fork’	If there is no partial dependency (best parents or witnesses), then transactions are eliminated from the MCI (tiebreaker rule)
Fault Tolerance	YES	YES 2n/3 (N: total number of consensus participants)	Not defined
Immutability	By assigning weight to every transaction after every single direct or indirect conformation and the MCMC random walk selection makes it impossible to move back on Tangle and change previous transactions	Strong seeing protocol	Storing own hash and parents hash (cryptographically linked)
**DAG and IoT**			
Resource requirement	No specialized hardware requirement	No specialized hardware requirement	No specialized hardware requirement
Applicable to IoT	Micropayments and distributed ledger for resource-imitated IoT systems	Micropayments and virtual voting reduce the system overhead of network communication	Only storing hashes of the data makes it suitable for low storage devices
**Generic Characteristics**			
Throughput	No Upper bound	2.5 × 10^5^ TPS	Not defined
Distinctive features	Nonlinear structure. In contrast to the legacy blockchain, the scalability increases as the number of transactions increase	Fairness of order, timestamp of order	Nonlinear (SKIPLIST) DAG Data structure
Limitations	Coordinator makes it decentralized (although L2 chains and enhancements are promising in the future). If the transaction rate is low, then the unforeseen new transaction confirmation is delayed	Possibility of larger communication delays due to the gossip protocol, variants of Hashgraph, and different vertices	Due to witnesses, tends toward centralization and is not entirely open-source

## Data Availability

The formal representation of ontologies in RDF/OWL format can be downloaded here https://cloudstor.aarnet.edu.au/plus/s/OvN6Wtr8ErxCHRZ, accessed on 17 October 2022, https://cloudstor.aarnet.edu.au/plus/s/4tTu0417VgJfTOo, accessed on 17 October 2022.
